# Neurofilament Light Chain and Cognitive Performance in Selected Siblings From the CATSLife Study

**DOI:** 10.1093/geroni/igaa057.398

**Published:** 2020-12-16

**Authors:** Chandra Reynolds, Andrew Smolen, Christopher Link, Sally Wadsworth

**Affiliations:** 1 University of California, Riverside, Riverside, California, United States; 2 University of Colorado Boulder, Boulder, Colorado, United States; 3 University of Colorado, Boulder, Colorado, United States

## Abstract

Markers of neurodegeneration such as neurofilament light chain (NfL) may be elevated with neurological diseases such as multiple sclerosis (MS) as well as Alzheimer’s disease. NfL is a marker of axonal integrity where higher values positively relate to the degree of damage. NfL shows variations in early adulthood among healthy individuals and may relate to executive function performance in otherwise healthy individuals aged 19-32 years. In the ongoing CATSLife (Colorado Adoption/Twin Study of Lifespan behavioral development and cognitive aging) Quanterix Simoa assays of NfL were measured in 34 individuals selected based on self-reported neuroinflammatory conditions (N = 5) or by APOE genotype (N_nonE4 = 18, N_E4 = 16). The distribution of NfL was consistent with other studies of early-mid adulthood (range = 1.3 - 22.3 pg/ml). Based on partial regression weights predicting log-transformed NfL, NfL was higher in cases (exp(b)=1.08 pg/ml), in males (exp(b)=1.25 pg/ml), by age (exp(b)=1.03 pg/ml per year) and in APOE E4 carriers (exp(b)=1.11 p/mg). Moreover, correlations partialed for age, sex, APOE e4 and case status suggest higher NfL may be associated with lower Full Scale IQ and general cognitive ability (r’s = -.18 and -. 28) overall and may be more evident among APOE E4 carriers (r’s = -.42 - .44, partialed for age, sex, case status). In this pilot study, the observed NfL associations with general cognitive ability, particularly among APOE E4 carriers, suggests NfL may be a salient biomarker of cognitive functioning by early- to mid-adulthood.

